# The Effectiveness of Traditional Chinese Medicine in Treating Alzheimer’s Disease

**DOI:** 10.1192/bjo.2025.10112

**Published:** 2025-06-20

**Authors:** Collin Chong, Sian Edney, Benjamin Jelly

**Affiliations:** Cardiff University, Cardiff, United Kingdom

## Abstract

**Aims:** The prevalence of Alzheimer’s disease (AD) is increasing across the world, yet extensive research has not yielded effective curative treatment. Traditional Chinese Medicine (TCM) has shown benefits and potential in treating AD. However, there were no recent systematic reviews of the different TCM modalities in treating AD specifically. The primary aim of this systematic review (SR) was to investigate the effectiveness of TCM either as a standalone or adjunct treatment alongside conventional medication for AD. The secondary aim was to provide recommendations for treating AD by exploring the addition of TCM in clinical practice.

**Methods:** A systematic review with meta-analysis was performed, following Preferred Reporting Items for Systematic reviews and Meta-Analyses (PRISMA) guidelines. A search was performed on Medline, Allied and Complementary Medicine Database (AMED), Web of Science and Scopus, while grey literature was searched on Overton. There were no filters set on the date of publication, language, subject and author. The search terms of Traditional Chinese Medicine and Alzheimer’s disease were searched till 27 July 2024. The studies were screened to target patients with AD, therapies and herbs that have corresponding names in TCM and are randomised controlled trials (RCTs). This led to eleven included RCTs for narrative review and meta-analyses. Study Risk of Bias Assessment and quality assessment were performed. A random effects model was used in the meta-analysis to assess the efficacy of treatments.

**Results:** A total of 11 randomised controlled trials that comprise 1155 patients with AD were included. The included studies showed that TCM therapies of saffron, cistanches herba, MLC601, and Shenfu or Shenmai with Deproteinized Calf Blood Injection (DCBI) had therapeutic benefits as stand-alone therapy for improving cognitive function in patients with AD. Combined with results from the meta-analyses, bushenhuatanyizhi, Guilingji and Jiannao Yizhi formula also showed potential as stand-alone therapy in improving cognitive function and activities of daily living. Two studies supported adding Dengzhan Shengmai or Huanglian Jiedu decoction as an adjunct to conventional treatment. Hachimijiogan showed some effect in slowing cognitive decline as an adjunct therapy, while Yokukansan can help with managing behavioural and psychological symptoms of dementia.

**Conclusion:** This review adds support to the current literature of the potential of TCM therapies in treating AD. A search on the Chinese databases, investigating the mechanisms behind how these interventions work and using a combination of TCM therapies are potential future studies on alternate forms of treatment for patients with AD.

